# Odd-even oddball task: Evaluating event-related potentials during word discrimination compared to speech-token and tone discrimination

**DOI:** 10.3389/fnins.2022.983498

**Published:** 2022-10-14

**Authors:** Marcus Voola, An T. Nguyen, Welber Marinovic, Gunesh Rajan, Dayse Tavora-Vieira

**Affiliations:** ^1^Division of Surgery, Medical School, The University of Western Australia, Perth, WA, Australia; ^2^Department of Audiology, Fiona Stanley Fremantle Hospitals Group, Perth, WA, Australia; ^3^School of Population Health, Curtin University, Perth, WA, Australia; ^4^Department of Otolaryngology, Head and Neck Surgery, Luzerner Kantonsspital, Luzern, Switzerland

**Keywords:** oddball paradigm, event related potential (ERP), P3b, EEG, auditory, semantic

## Abstract

Tonal and speech token auditory oddball tasks have been commonly used to assess auditory processing in various populations; however, tasks using non-word sounds may fail to capture the higher-level ability to interpret and discriminate stimuli based on meaning, which are critical to language comprehension. As such, this study examines how neural signals associated with discrimination and evaluation-processes (P3b) from semantic stimuli compare with those elicited by tones and speech tokens. This study comprises of two experiments, both containing thirteen adults with normal hearing in both ears (PTA ≤ 20 dB HL). Scalp electroencephalography and auditory event related potentials were recorded in free field while they completed three different oddball tasks: (1) tones, (2) speech tokens and (3) odd/even numbers. Based on the findings of experiment one, experiment two was conducted to understand if the difference in responses from the three tasks was attributable to stimulus duration or other factors. Therefore, in experiment one, stimulus duration was not controlled and in experiment two, the duration of each stimulus was modified to be the same across all three tasks (∼400 ms). In both experiments, P3b peak latency was significantly different between all three tasks. P3b amplitude was sensitive to reaction time, with tasks that had a large reaction time variability resulting in the P3b amplitude to be smeared, thereby reducing the amplitude size. The findings from this study highlight the need to consider all factors of the task before attributing any effects to any additional process, such as semantic processing and mental effort. Furthermore, it highlights the need for more cautious interpretation of P3b results in auditory oddball tasks.

## Introduction

Event Related Potentials (ERPs) capture the cortical responses after the presentation of auditory stimuli, which can be in the form of tones, speech tokens, or words (Balkenhol et al., 2020). Recording the brain activity in response to auditory stimuli provides a method for understanding how the brain processes various auditory input and what factors are important for sound processing.

ERPs can be divided into two categories: exogenous and endogenous potentials. Exogenous potentials (P1, N1, P2, and N2) are pre-attentive responses and can be elicited automatically in the response to the stimulus (i.e., the participant does not have to physically respond to a particular stimulus). These typically occur within the first 250 ms of stimulus onset and are thought to not reflect cognitive processing (Martin et al., 2008; Lightfoot, 2016; Tao et al., 2022). Endogenous potentials, such as the P300, are theorized to reflect attention and working memory because they require the participant to respond to a particular change in stimuli characteristic, e.g., intensity and/or frequency (Polich, 2007). The P300 can be divided into two components: the P3a and P3b, with both components occurring within a latency range of 300–600 ms after stimulus presentation. The P3a can be differentiated from the P3b because it has a shorter peak latency, it is generated from the cingulate, frontal areas of the brain, and is thought to reflect involuntary allocation of resources to a change in stimuli (Volpe et al., 2007; Kamijo, 2016). Conversely, the P3b is thought to quantify an individual’s ability to discriminate and interpret auditory stimuli (Polich, 2007). The P3b is characterized by a parietally distributed positivity and can be localized to the parietal and temporo-occipital regions of the brain (Volpe et al., 2007).

One method to elicit the P3b is by using an oddball paradigm, which consists of two types of stimuli: an infrequent (target) stimulus and a frequent (standard) stimulus, whereby the target stimuli are characterized by a unique feature (Polich, 2007). The P3b positivity is enhanced upon the presentation of the target stimuli in the oddball paradigm when compared with the presentation of the standard stimuli (Didoné et al., 2016). The P3b has been suggested to reflect the decision-making ability of an individual and subsequent activation of stimulus response links (Verleger, 2020). If this decision-making hypothesis is true, then it is expected that during more complex tasks, when decisions demand greater higher order processing, P3b amplitude will be larger and latency will be delayed (Polich, 2007; Kelly and O’Connell, 2013). The link between P3b and decision making is further supported by the alignment of the P3b deflection to the onset of the response (Sassenhagen et al., 2014).

Studies using P3b have looked at discrimination of tones (Ana Paula et al., 2016; Kalaiah and Shastri, 2016), but more studies have used more complex word-like stimuli (Kotchoubey and Lang, 2001; Balkenhol et al., 2020), arguing that the latter may be more suitable for language research because they are more naturalistic and contain semantic aspects which are missing from tones (Verleger et al., 2014; Li et al., 2019). Speech tokens have also been used to investigate individuals’ higher order processing capabilities. Similar to the establishment of tonal stimuli, oddball paradigms incorporating speech tokens have been adapted to be more complex by using speech tokens that have a close phonetically similarity. The P3b of speech tokens that were more phonetically similar (i.e., /ba/vs/ga/are more phonetically similar than/ba/vs/di/), thereby making the participant attend more closely to identify the target stimuli. This resulted in P3b latency being delayed and of larger amplitude. These findings highlight that a delayed and enhanced (more positive) P3b may suggest that sounds which are phonetically similar require more time and cognitive resources to discriminate (Didoné et al., 2016).

Tonal and speech-token tasks have been commonly used in the past; however, they fail to capture the ability to interpret and discriminate stimuli based on meaning. This is attributed to both tasks involving the discrimination of the physical characteristics of the sound (e.g., frequency and fast-changing temporal qualities associated with articulation). As such, if the decision-making hypothesis is true, it is theorized that the use of words (semantics) in oddball paradigms requires participants to differentiate on the physical properties and on the semantic meaning of the sound. A tonal and speech token oddball task can reveal auditory deficits, but the lack of complexity in the task (it only requires participants to differentiate based on physical properties) may result in some auditory processing deficits not being detected (Henkin et al., 2009; Finke et al., 2016; Balkenhol et al., 2020).

Words have been used in oddball paradigms to elicit these ERPs. In 2016, Finke et al. used an oddball paradigm that required subjects to semantically classify words as either living or non-living entities (Finke et al., 2016). This rationale was derived from the decision making hypothesis, as it is believed that greater stimulus complexity, in the form of differentiating stimuli based on meaning rather than just physical properties, may result in more complex neural circuitry being engaged. These additional circuits include retrieving word meanings from our mental lexicon and the circuits involved in categorizing words based on these meanings, which will be reflected in the P3b by delayed latency and larger amplitude (Henkin et al., 2009; Finke et al., 2016; Balkenhol et al., 2020). However, this rationale is not supported by Kotchoubey and Lang (2001), who compared the P3b latency and amplitude from participants with normal hearing in two different oddball tasks: a classical tonal oddball task and semantic oddball task. Participants had to count the number of times an “animal” name was presented in a series of nouns that could be classified into four separate categories (tools, jobs, body parts, and household objects). Kotchoubey and Lang also identified a delayed P3b latency but found a smaller P3b amplitude in the semantic oddball task when compared to the classic tonal oddball task (Kotchoubey and Lang, 2001). This finding is interesting because it suggests that semantic oddball tasks may not produce P3b results that are consistent with the decision making hypothesis that has been used in previous studies (Henkin et al., 2009; Finke et al., 2016; Balkenhol et al., 2020) and highlights that other factors, such as stimulus complexity (i.e., number of stimuli), may contribute to the P3b response.

### Current study

This study is comprised of two experiments, both of which aim to develop a semantic oddball task which requires participants to discriminate sounds based on their meaning rather than just their physical properties. In addition, both experiments aim to examine how neural signals associated with discrimination and evaluation-processes (P3b) from semantic stimuli compare with those elicited by tones and speech-like sounds (speech tokens). We will be comparing the size of the semantic evoked ERPs with simpler and already established auditory oddball paradigms, tones, and speech tokens. To the best of our knowledge, no study has investigated the differences in P3b responses between tonal, speech token, and semantic oddball paradigms in the same experiment. In addition, there is little research describing the P3b in response to meaningful word stimuli. Past studies have theorized the differences between tonal, speech token, and oddball tasks and nobody has investigated if these theorized differences are indeed true. Understanding the differences (if any) in the neural processes between physical and semantic tasks may provide auditory scientist with more information about the task they choose to use for research and the factors they need to consider when designing an experimental task.

It is hypothesized that the additional processing required to discriminate between the semantic odd/even tasks will result in a larger and delayed P3b when compared with the tonal and speech token task. Upon analysis of the results of the first experiment, we identified that while there was a difference between the three tasks, these differences could be attributed to the lack of control for stimulus duration and critical latency rather than a semantic component. Critical latency was defined as the earliest time point after stimulus-onset when a stimulus can be uniquely identified. For example, this was the first point in time participant would be able to differentiate/ba/from/da/or “two” from “three.” To understand if the differences between tasks that were identified in experiment one are attributed to stimulus duration, experiment two was developed to (1) validate these findings and (2) control for stimulus duration.

## Materials and methods

This study consisted of two separate experiments. In both experiments, each participant completed three versions of the acoustic oddball task (tonal, speech-token, and odd-even numbers). In experiment 1, the duration of each stimulus was not controlled (i.e., tones, speech tokens, and odd-even numbers all had the same stimulus duration). However, in experiment 2, stimulus durations were adjusted to correct for differences across the three tasks. In the following sections, both experiments will be described together, and distinctions will be highlighted in the relevant sections.

### Participants

Participants were healthy adult volunteers (students and clinicians) recruited from the Audiology department at Fiona Stanley Hospital (Perth, Australia). Experiment 1 consisted of thirteen participants [Mean(SD)_*age*_ = 25.20(3.89) years, 6 females, 7 males]. Experiment 2 also consisted of thirteen participants [Mean(SD)_*age*_ = 25.3(3.79) years, 5 females, 8 males]. Four participants completed both experiments. All participants had their hearing thresholds tested and were all within the normal hearing range (less than 20 dB HL in all frequency ranges from 0.5 Hz to 8 kHz in both ears). The mean (SD) unaided air pure tone average (PTA) from 0.5 Hz to 8 kHz in experiment 1 was 4.95 ± 3.60 dB HL in the left ear and 4.61 ± 2.92 dB HL in the right ear. In experiment 2 the PTA was 6.15 ± 2.41 dB HL in the left ear and 5.29 ± 2.13 dB HL in the right ear. All participants provided written informed consent before the beginning of the experiment. Ethics approval for this study was obtained from the South Metropolitan Health Ethics Committee (Approval Code: 335).

### Procedure

**FIGURE 1:**
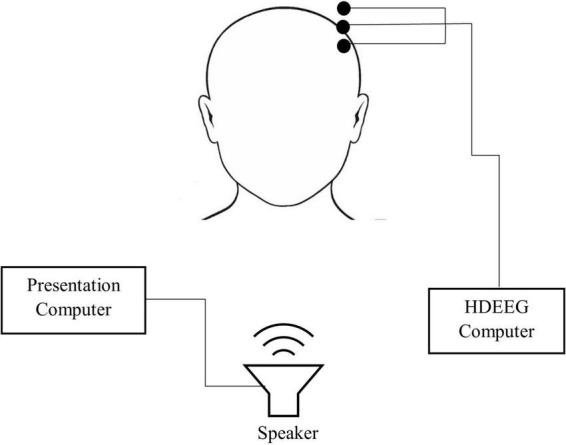
Schematic diagram of the set up used in both experiment 1 and 2. Testing was conducted in a sound attenuated booth.

For both experiments, participants were seated facing a wall in a dimly lit sound-attenuated room. Participants were fitted in an EEG cap and were provided with verbal instructions before completing three experimental tasks (see Experimental Tasks section). Participants were instructed to keep their eyes open throughout the experiment. Self-paced breaks were taken between tasks. Task order was counter-balanced across participants for each experiment. The entire experiment took around 60 min, which included informed consent, hearing assessment, preparation of the participant and the three oddball tasks (tones, speech tokens and odd-even). Each oddball task took approximately 8 min to complete. Figure 1 shows a schematic diagram of the experimental set up.

### Experimental tasks

Each experiment consisted of three acoustic oddball tasks with different stimuli (tones, speech tokens, and odd-even numbers). In each task, participants completed 240 trials where they were presented with an acoustic “standard” or “target” stimulus. Their task was to respond as soon as they heard the target stimulus by pressing a button on the response pad with their right thumb. Standard and target stimuli were presented on 80% (192) and 20% (48) of trials, respectively, and trials were presented pseudo-randomly such that a target stimulus would not be presented on two consecutive trials. All stimuli were presented in free-field at a calibrated intensity of 55–60 dB SPL using EDIFIER M1250 Multimedia Speakers, with an inter-stimulus interval of 1,800 ms. Cogent 2000 and Psychtoolbox-3 functions in MATLAB were used to control the experiment. Reaction time was the time of button press relative to stimulus onset.

The pure tone and speech-token tasks each consisted of two stimuli and the odd-even numbers task consisted of eight stimuli. In the pure tone task, 1 and 2 kHz pure tones were used. In the speech-token task, synthesized/ba/and/da/sounds were used. These speech-tokens were obtained from National Acoustic Laboratories (NAL) and were first used by Johnson et al. (2008). In the odd-even numbers task, the pre-recorded numbers 1, 2, 3, 4, 5, 6, 8, and 9 were used. The number seven was omitted because it contains two syllables. The eight numbers were grouped into odd numbers (1, 3, 5, 9) and even numbers (2, 4, 6, 8), creating two different stimuli groups. The target stimuli for each of the three tasks was counterbalanced amongst participants. As such, participants were instructed to respond to either 1 or 2 kHz for the pure-tone task, either/ba/or/da/for the speech-token task and either odd or even numbers for the odd-even number task. Participants who completed both experiment one and two were not given the same target stimuli between experiments one and two. This was done to account for any learning effects.

These recordings were obtained from NAL and were spoken by a mature female Australian English speaker. These speech files were recorded to be used in a telephone-based speech-in-noise test called “Telescreen” (Dillon et al., 2016). These recordings were modified in Audacity to reduce the discrepancy in stimulus duration between numbers while maintaining intelligibility. In experiment 1, the duration of tones was 100 ms, the duration of speech-tokens 170 ms, and the average duration of odd-even numbers was 400 ms. In experiment 2, the duration of tones and speech-tokens were lengthened using Audacity to match average duration odd and even numbers. The spectral content of the pure tone and speech tokens was not modified, rather the existing sound file was extended by replicating the sound wave so that the audio lasted for 400 ms.

### Acquisition and pre-processing of electrophysiological data

Electrophysiological data were continuously recorded for the duration of each task. Data were acquired using the Micromed™ SD LTM EXPRESS system (Treviso, Italy), a SpesMedica cap (Genoa, Italy) and Gilat Medical ERP software (Karkur, Israel), at a sampling rate of 1,024 Hz with an online low pass-filter of 40 Hz. Data were recorded from 59 Ag/AgCl scalp electrodes arranged according to the 10–20 system with additional electrodes placed under the right infraorbital region to monitor eye-movement. A reference electrode placed on the middle of the chin and a ground electrode was placed on the right mastoid. All electrode impedance was kept below 5 kΩ for the duration of the recording.

MATLAB 2020a was used to process the data. A semi-automated procedure was used consisting of functions from the plug-ins EEGLAB (Delorme and Makeig, 2004), PREP pipeline (Bigdely-Shamlo et al., 2015), clean_rawdata() plugin, AMICA (Palmer et al., 2011), and ICLabel plugin (Pion-Tonachini et al., 2019). The removeTrend() from the PREP pipeline plugin was used to linearly detrend the data using a high-pass 1 Hz FIR filter (step size = 0.02). The cleanLineNoise() from PREP pipeline plugin was used to remove 50 Hz line noise and harmonics up to 500 Hz. The pop_clean_rawdata() was used to determine noisy channels. The pop_interp was used to interpolate noisy channels spherically. EEG data was then down-sampled to 250 Hz. The data was demeaned and a 30 Hz low-pass filter (order = 100) was applied using the pop_eegfiltnew(). The clean_asr() was used to correct for artifacts using the artifact subspace reconstruction method (*SD* = 100).

Initially, the data were epoched from −200 to 1,000 ms relative to stimulus-onset. However, further investigation highlighted that task-effects may be driven by a difference in “critical latency,” which is the earliest point after stimulus-onset when a stimulus can be uniquely identified and distinguished. For example, this was the first time point a participant would be able to differentiate/ba/from/da/or “two” from “three.” Two individuals with normal hearing determined the critical latency and were instructed to indicate the earliest time at which they could identify which tone, speech token, or odd/even number was being presented. After averaging the results from both individuals with normal hearing, the critical latency could be determined. For tones, the critical latency was 0 ms (i.e., at stimulus onset). For speech tokens, the critical latency was 40 ms for both/ba/and/da/. For odd-even numbers, the critical latency ranged from 30 to 250 ms (one = 150 ms, two = 125 ms, three = 30 ms, four = 250 ms, five = 200 ms, six = 40 ms, eight = 60 ms, and nine = 140 ms).

As such, we also extracted −400 to 1,000 ms epochs time-locked to the critical latency of each stimulus, thereby discarding the data extracted from −200 to 1,000 ms. Independent component analysis (ICA) of the data was conducted using AMICA (2000 iterations) on down sampled data to 100 Hz (Palmer et al., 2011). The number of independent components extracted were adjusted for the data rank. ICLabel functions were used to classify and remove independent components that were eye movement, muscle, heart, line noise, or channel noise with >70% confidence. The data were baseline correct to the pre-stimulus interval. Trials with activity exceeding 100 μV were excluded from further analysis.

### Measurement of event-related potentials

We measured the mean amplitude of P3b at the single trial level on waveforms time-locked to stimulus-onset and the critical-point. P3b amplitude was measured at the centroparietal mid-line electrode (CPz) where the P3b was most prominent. P3b was also measured over a 40 ms interval around the positive peak of the grand average waveform between 300 and 600 ms.

Peak latency of the P3b was measured at the level of the participant’s grand average waveform and time-locked to both stimulus-onset and the critical-point. Measurements at the level of the participant were undertaken after single trial level measurements resulted in large variability for peak latency. P3b peak latency was measured as the time-point of maximum amplitude between 300 and 800 ms at CPz.

### Reaction time and reaction time variability

Reaction time (RT) was the duration between target stimulus onset and participant response. Furthermore, this was the time taken for the participant to identify and categorize a stimulus as a target stimulus. To account for differences in critical latency between the three oddball tasks, adjusted reaction time was calculated at a trial level by subtracting the critical latency for the presented target stimuli from the participant’s reaction time. Reaction time variability was calculated by averaging the standard deviation of the participant’s reaction time for each task.

### Stimulus and response-aligned event related potential image

To illustrate the relation between P3b, stimulus-onset, and reaction time, we generated ERP image plots depicting single trial EEG activity focusing on the P3b effect (Target-Standard difference waveforms at CPz). The EEG data plotted represents a pool of trials from all participants, which were sorted by reaction time and plotted as a colormap (blue = negative, red = positive). Trial level difference waveforms were achieved by subtracting the grand-average standard ERP from each target trial waveform. We also indicated the time of stimulus and response-onset by overlaying black solid and red dashed lines, respectively.

In the ERP image, we represented the data in two ways: “stimulus-locked” and “response-locked.” For stimulus-locked, the ERP data were aligned to stimulus-onset (illustrated by the vertical black line at 0 ms on the top-half of the image). RT varies across trials (illustrated by the curved red line ∼400 ms). Most notably, the positivity in the EEG signal (shown by the red of colormap) roughly coincides with RT. In other words, the P3b activity seems to show a response-alignment. To further highlight this point, we used “response-locked” plots which re-aligns the same EEG data to time of response-onset (illustrated by the vertical lines between 350 and 600 ms on the bottom-half of the image). In this plot, positivity is no longer “smeared” over time; instead, it is clustered around the red dashed line, which illustrates that the P3b shows a response-alignment.

Under the ERP image are two grand-averaged waveforms computed from the stimulus and response-locked ERP data. For Speech-Token and Odd/Even tasks, the amplitude of stimulus-locked data is smaller than response-locked data because the timing of the P3b is more varied in the stimulus-locked data. This illustrates that differences in P3b amplitude are a by-product of differences in RT variability as opposed to a true difference in amplitude across tasks.

### Statistical analysis

All statistical analysis was conducted using R statistics and R software. A linear mixed model analysis was employed using “lmer” function from the “lmerTest” package. For the N2N4 analysis we modeled the amplitude with trial type, task and the interactions have fixed effects, with participant intercepts modeled as a random effect. For P3b we modeled amplitude using the same method. The results of the model were presented as F values using the Satterthwaite’s approximation method with the “ANOVA” function. We used “r2beta” to calculate the effect size. Main effects and interactions were evaluated by comparing the estimated marginal means using the “emmeans” function from the “emmeans” package. The results of these pairwise categorical comparisons were presented as t-ratios (mean difference estimate divided by standard error) with degrees-of-freedom estimated using the Kenward-Roger method.

## Results

### Overview

Results from both experiment 1 and 2 are presented herein. The P3b peak amplitude and latency figures are presented to illustrate the difference observed between the three different oddball tasks. These illustrations are repeated twice for each experiment: once looking at the effects when time locked to onset latency and once looking at the effects when time locked to critical latency. In addition, differences in reaction time between the three tasks for both experiments are depicted. Overall, the results of both experiments suggest that the difference between the Odd/Even oddball task when compared to the speech token and tonal oddball task can be attributed to either physical differences in the stimuli (critical latency and stimulus duration) or a semantic component.

### Reaction time

**FIGURE 2:**
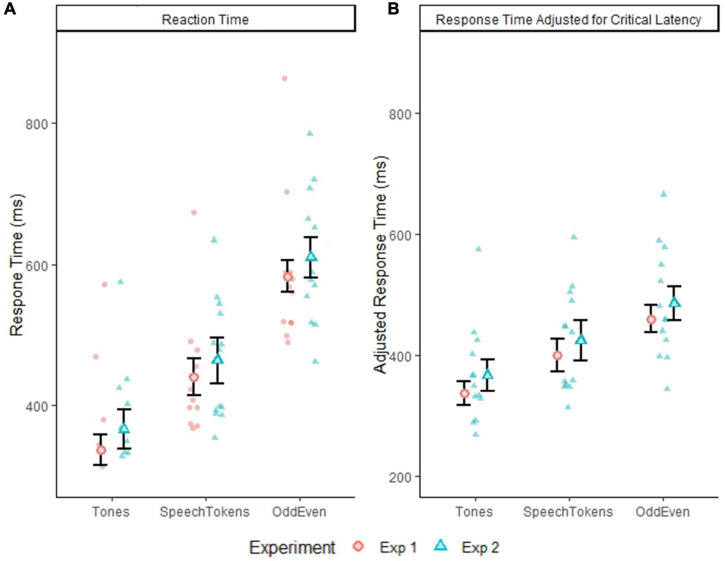
**(A)** Depicts the group average reaction time with within subject error bars for all three tasks from experiment 1 and 2. **(B)** Depicts the group average reaction time adjusted for critical latency. Error bars reflect standard error of the mean. The smaller faded red circles **(Experiment 1)** and smaller faded blue triangles **(Experiment 2)** represent the individual subject averages for each task.

The reaction times for all three oddball tasks across both experiments are displayed in Figure 2A. Pairwise comparisons identified a significant difference for comparisons between all three tasks: tones vs. speech tokens [*t*(3692) = −24.164, *p* = < 0.0001], speech tokens vs. Odd/Even [*t*(3692) = −34.669, *p* = < 0.0001], and tones vs. Odd/Even [*t*(3692) = −58.860, *p* = < 0.0001]. Reaction time was also calculated considering the difference in critical latency between conditions (Figure 2B). Despite accounting for differences in critical latency, pairwise comparison of all three oddball tasks revealed a significant difference between all three tasks: tones vs. speech tokens [*t*(3692) = −13.892, *p* = <0.0001], speech tokens vs. Odd/Even [*t*(3692) = −13.928, *p* = <0.0001], and tones vs. Odd/Even [*t*(3692) = −27.826, *p* = <0.0001].

### P3b latency

**FIGURE 3:**
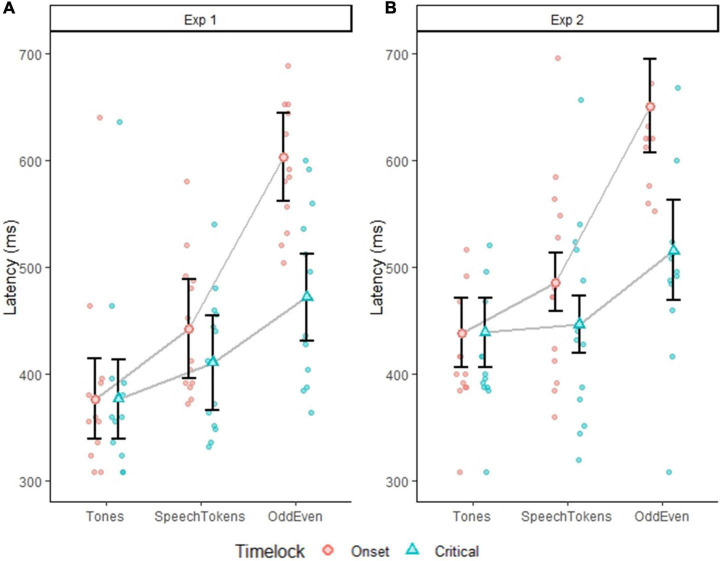
Plots depict the average P3b peak latency for all three tasks average on a subject level. Error bars represent the standard error of the mean. **(A)** Depicts the difference in P3b peak latency for experiment 1 when time locked to onset (red dots) and critical latency (blue dots). **(B)** Depicts the difference in P3b peak latency for experiment 2 when time locked to onset (red dots) and critical latency (blue dots). Faded red and blue dots represent each subject P3b latency.

In experiment 1, P3b peak latency, when time locked to onset latency, identified significantly different P3b peak latencies in all three oddball tasks: [*t*(60) = 37.341, *p* = <0.0001] (Figure 3A): tones vs. speech tokens [*t*(24) = −2.222, *p* = 0.0360], speech tokens vs. Odd/Even [*t*(24) = −5.445, *p* = <0.0001], and tones vs. Odd/Even [*t*(24) = −7.666, *p* = <0.0001]. When time locked to critical latency, a significantly shorter P3b peak latency was observed only for tones when compared with Odd/Even [*t*(24) = −3.286, *p* = 0.0094].

When stimulus duration was controlled for in experiment 2, P3b peak latency was still when time locked to onset latency was significantly delayed for Odd/Even when compared with both tones [*t*(24) = −8.753, *p* = <0.0001] and speech tokens [*t*(24) = −6.799, *p* = <0.0001] (Figure 3B). When time-locked to critical latency, similar effects were observed except the magnitude of the difference was reduced. P3b peak latency for Odd/Even was still significantly delayed when compared with tones [*t*(24) = −7.666, *p* = 0.0173] and speech tokens [*t*(24) = −2.727, *p* = 0.0235].

### P3b amplitude

**FIGURE 4:**
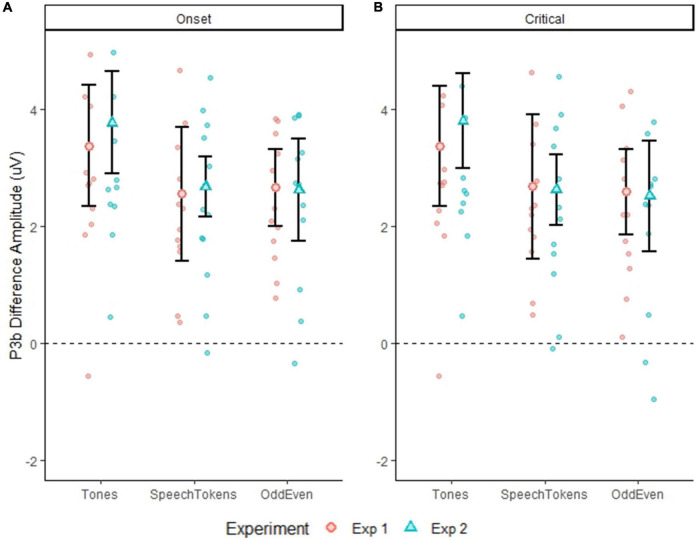
Differences in P3b amplitude for both experiments for all three tasks calculated at a single trial level. Error bars represent standard error of the mean. **(A)** Shows the difference in the three tasks for both experiments when time locked to onset latency. **(B)** Shows the difference in the three tasks for both experiments when time locked to critical latency. The smaller faded red circles **(Experiment 1)** and smaller faded blue triangles **(Experiment 2)** represent the individual subject averages for each task.

P3b amplitude time-locked to stimulus onset and averaged over both experiments elicited a significantly larger amplitude for tones when compared with speech tokens [*t*(3477) = 4.934, *p* = <0.0001] and Odd/Even [*t*(3476) = 4.782, *p* = <0.0001]. Likewise, P3b amplitude time-locked to critical latency indicated a significantly larger amplitude for tones in comparison with speech tokens [*t*(3445) = 4.700, *p* = <0.0001] and Odd/Even [*t*(3444) = 5.334, *p* = <0.0001]. Figures 4A,B illustrate the P3b waveform morphology of both experiments and time-locks.

### Reaction time variability and P3b response-alignment

**FIGURE 5:**
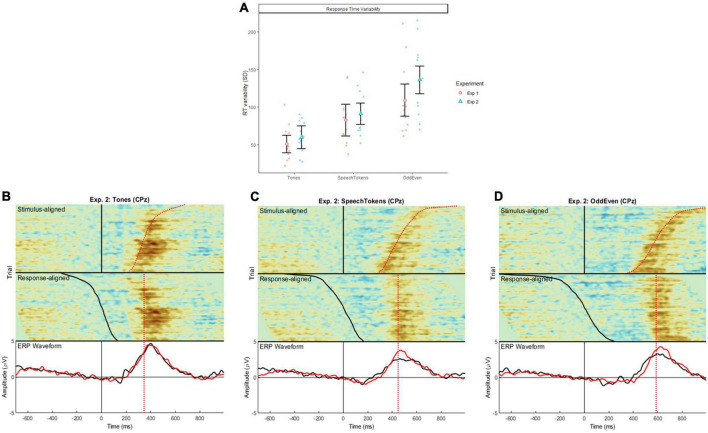
**(A)** Illustrates the difference in reaction time variability for the three tasks in both experiments 1 and 2 calculated at a single trial level. Error bars represent standard error of the mean. Shaded red circles and blue triangles represent the average reaction time variability for each subject. **(B–D)** Show the stimulus aligned waveform (i.e., time locked to stimulus onset) and response aligned (i.e., time locked to the subject’s reaction time) for tones, speech tokens, and Odd/Even, respectively. The black ERP waveform represents the waveform generated from the stimulus aligned data; the red ERP waveform represents the response aligned data.

Reaction time variability was also calculated to examine the response-alignment of the P3b signals across the three tasks and both experiments (Figure 5A). Critical latency of target trials was subtracted from the participant’s reaction time at a trial level. Reaction time variability was significantly smaller for tones when compared with speech tokens [*t*(48) = −3.720, *p* = 0.0005] and Odd/Even [*t*(48) = −8.485, *p* = <0.0001]. Reaction time variability to speech token was significantly smaller than to Odd/Even [*t*(48) = −4.765, *p* = <0.0001].

**FIGURE 6:**
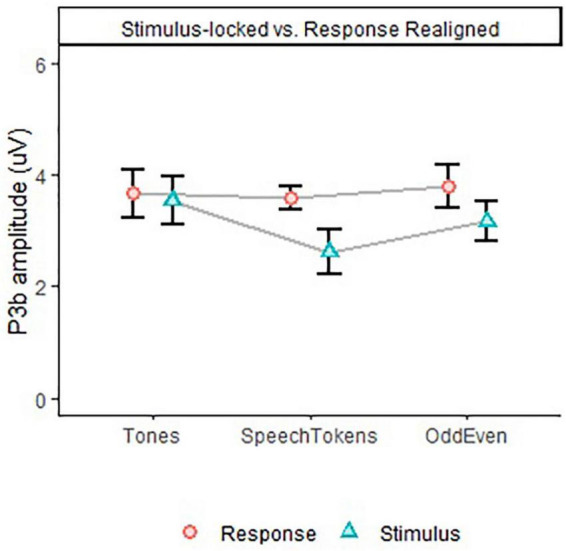
P3b amplitude for all three tasks in Experiment 2, depicting differences in task effect on stimulus and response aligned data. Error bars represent standard error of the mean.

Comparison of stimulus and response aligned ERPs are illustrated in Figures 5B–D for tones, speech tokens and Odd/Even respectively. Response aligned ERPs indicate that the P3b amplitude for speech tokens and Odd/Even was suppressed due to the high reaction time variability. On the other hand, the small reaction time variability in the tonal task elicited a larger P3b due to more consistent trial to trial responses. We analyzed the effect of task on the stimulus and response aligned P3b amplitude data (Figure 6) and identified that the significant effect of task in the stimulus aligned data [*F*(2, 1691.4) = 4.05, *p* = 0.018] was no longer present when the response data are aligned [*F*(2, 1691.2) = 0.40, *p* = 0.668].

## Discussion

It has been argued that tonal oddball tasks lack the semantic components are critical to language processing, therefore making them of questionable suitability for real world testing (Schirmer et al., 2005). In addition, there is inconsistency in the literature, with some studies hypothesizing that using semantic oddball tasks may engage additional semantic processing, which might enhance the associations between ERP responses and measures of language and comprehension (Henkin et al., 2009; Finke et al., 2016; Balkenhol et al., 2020); and other literature indicating that there is no difference between tonal and semantic oddball tasks (Kotchoubey and Lang, 2001). To address these conflicting claims, the present study had two aims: firstly, to develop a semantic oddball task which forced participants to discriminate auditory stimuli based on meaning rather than on differences in the physical properties of the sound, and secondly, to compare this task against more established oddball paradigms that use tonal and speech token stimuli. We hypothesized that with increasing stimulus complexity, reaction time would increase, P3b latency would be delayed, and amplitude would be enhanced.

In experiment 1, we identified an increase in reaction time and P3b latencies for increased stimulus complexity (tones < speech tokens < odd/even). Amplitude of P3b did not follow the same trend. Rather, it was larger for simple stimuli (tones) when compared with complex stimuli (speech tokens and odd/even numbers). The difference in critical latency and stimulus duration between the three oddball tasks could be an explanation for why this was observed.

The critical latencies of the three tasks were not the same: in tones it was 0 ms, in speech tokens it was 40 ms, and in odd/even it was 60–250 ms. This variation is explainable. Participants could differentiate between tones as soon as they heard the stimulus because it only requires them to detect the difference in frequency. Participants can quickly differentiate speech tokens from tonal stimuli when they perceive the/d/or/b/phoneme. On the other hand, differentiating between odd and even numbers takes longer. Taking the numbers “four” and “five” as an example, both numbers begin with a/f/phoneme, so even though a participant detects this sound they still must wait for more auditory information before they can differentiate the two numbers. To account for these differences, the data from experiment 1 were re-analyzed time-locked to the critical latency. When aligned to critical latency there was no significant impact on the reaction time, P3b latency, and P3b amplitude with the same trend in results being observed. The size of the difference was reduced but the effect was still significant.

The second possible explanation for the trend in results was the difference in stimulus duration across all three oddball tasks. This rationale is supported by previous studies which identified that endogenous potentials such as ERPs, are affected by stimulus properties such as intensity, pitch, and stimulus duration (Alain and Winkler, 2012). This led to the development of experiment 2, which contained the same three oddball tasks as experiment 1 but the stimulus duration of the tonal and speech token oddball task was matched to the duration of the odd/even task. Despite the control of stimulus duration, we identified that the same trend in data from experiment 1. Reaction time, P3b peak latency, and P3b amplitude were all significantly different but just with the effect size being reduce when the data was time locked to critical latency. Neither critical latency nor stimulus duration had a significant effect on the results. This indicates that our results cannot be attributed to differences in critical latency and stimulus duration.

The enhancement of P3b amplitude for tones compared to speech tokens and odd/even was surprising. Our initial hypothesis was that the P3b amplitude would be enhanced with increasing stimulus complexity; however, we observed the opposite. Kotchoubey and Lang (2001) identified that a semantic oddball task had a delayed P3b latency and smaller amplitude when compared with a tonal oddball task. They suggested that the delayed latency between tonal and semantic oddball tasks may be attributable to the semantic oddball task having a greater number of stimuli compared to the tonal oddball task. Moreover, in the semantic oddball task there was 15 different target stimuli used per task (i.e., 15 different animal names), whereas in the tonal oddball task there was only one target stimuli. As a result, the stimuli from the semantic oddball tasks are more difficult to categorize. Despite this insight, there has been very little research done to understand how exactly stimulus complexity affects the P3b response (Kotchoubey and Lang, 2001).

To bridge this gap in the literature, we conducted further analysis to understand how stimulus complexity affects the P3b response. We identified that the large variability in reaction time could be an explanation for the P3b amplitude findings. As such, reaction time variability was calculated, which identified that variability was smallest in tones, then in speech tokens, and then largest in odd/even numbers. To account for these differences, data from experiment 2 were re-analyzed time-locked to the reaction time (Figure 5A). We found that then the P3b amplitude of both speech tokens and odd/even numbers increased. The P3b amplitude for tones stayed the same because there was very little difference in reaction time variability. These findings are in line with past studies which identified that variability in reaction time has a greater effect on P3b amplitude than P3b peak latency (Ramchurn et al., 2014; Verleger et al., 2014). Ramchurn et al. (2014) found that within recordings of the same subject, faster reaction times elicited a significantly larger P3b amplitude than slower reaction times. This accounts for the large variation in reaction time seen in the odd/even task: results in the effect of P3b amplitude were smeared out rather than concentrated in a peak (like in tones and speech tokens). This finding is supported by the significant differences in peak waveform morphology between the three tasks (Figure 5). Follow-up analysis identified that the effect is no longer significant when aligned to the reaction time of the participant (Figure 6).

The variability seen in the reaction time for the odd/even task could be attributed to the differences in the number of stimuli per task. More specifically, in the odd/even task there were four different targets and standards, each with a unique critical latency; whereas in the tones and speech token tasks, there was only one standard and target for each task. The greater variability in critical latency for the odd/even task is a possible explanation for the variability in reaction time. We hypothesize that this relationship exists because stimuli with a delayed critical latency will result in a participant requiring more time to differentiate the word, thereby requiring a longer reaction time. In the odd/even word list, there are eight different numbers, all with different critical latencies (range 30–250 ms). Given this large variability in critical latency, we hypothesize that this could be why there is larger variability in reaction time. Additionally, the spectral quality of the sound may also affect the reaction time variability, as poor sound quality may make it difficult for participants to identify and categorize auditory stimuli due to the lack of auditory information provided in the degraded signal. Future studies would benefit from controlling for the number of stimuli per task and ensuring the stimuli are of similar sound quality when comparing stimulus complexity.

Taken all together, the present study demonstrates that increasing stimulus complexity results in increased reaction time, reaction time variability, and P3b latency. We identified that critical latency and stimulus duration can only partly explain the effect of reaction time and P3b latency. Other factors such as differences in the spectral qualities of stimuli, semantic complexity, and the difference in the number of stimuli per task could also have contributed to the results. Additionally, variance in reaction time may help explain the P3b amplitude findings. Despite differences in stimuli, the morphology of the ERP waveforms was similar between tasks, which suggests that similar mechanisms are used between the tasks.

Our results highlight the importance of considering each aspect of the task before attributing effects to additional processes such as semantic processing and mental effort and, especially, amplitude. The study calls for more cautious interpretation of P3b results in semantic oddball tasks compared to simpler tasks. Future studies would benefit from examining the effect that the number of stimuli per task has on reaction time variability and P3b latency.

## Data availability statement

The raw data supporting the conclusions of this article will be made available by the authors, without undue reservation.

## Ethics statement

This study involving human participants were reviewed and approved by the South Metropolitan Health Ethics Committee (reference number: 335). The patients/participants provided their written informed consent to participate in this study.

## Author contributions

All authors listed have made a substantial, direct, and intellectual contribution to the work, and approved it for publication.
